# Dietary L-carnitine supplementation modifies blood parameters of mid-lactating dairy cows during standardized lipopolysaccharide-induced inflammation

**DOI:** 10.3389/fimmu.2024.1390137

**Published:** 2024-05-13

**Authors:** Leonie Seemann, Jana Frahm, Susanne Kersten, Susanne Bühler, Ulrich Meyer, Christian Visscher, Korinna Huber, Sven Dänicke

**Affiliations:** ^1^ Institute of Animal Nutrition, Friedrich-Loeffler-Institut, Federal Research Institute for Animal Health, Braunschweig, Germany; ^2^ Institute of Animal Nutrition, University of Veterinary Medicine Hannover, Hannover, Germany; ^3^ Department of Functional Anatomy of Livestock, Institute of Animal Science, University of Hohenheim, Stuttgart, Germany

**Keywords:** L-carnitine, dairy cow, mid-lactation, lipopolysaccharide, leukocyte functionality

## Abstract

L-carnitine, available as feed additive, is essential for the beta-oxidation of free fatty acids in the mitochondrial matrix. It provides energy to immune cells and may positively impact the functionality of leukocytes during the acute phase response, a situation of high energy demand. To test this hypothesis, German Holstein cows were assigned to a control group (CON, n = 26) and an L-carnitine supplemented group (CAR, n = 27, rumen-protected L-carnitine product: 125 g/cow/d, corresponded to total L-carnitine intake: 25 g/cow/d, supplied with concentrate) and received an intravenous bolus injection of lipopolysaccharides (LPS, 0.5 µg/kg body weight, *E. coli*) on day 111 *postpartum* as a model of standardized systemic inflammation. Blood samples were collected from day 1 *ante injectionem* until day 14 *post injectionem (pi)*, with frequent sampling through an indwelling venous catheter from 0.5 h *pi* to 12 h *pi*. All parameters of the white blood cell count responded significantly to LPS, while only a few parameters were affected by L-carnitine supplementation. The mean eosinophil count, as well as the percentage of basophils were significantly higher in CAR than in CON over time, which may be due to an increased membrane stability. However, phagocytosis and production of reactive oxygen species by leukocytes remained unchanged following L-carnitine supplementation. In conclusion, although supplementation with 25 g L-carnitine per cow and day resulted in increased proportions of specific leukocyte populations, it had only minor effects on the functional parameters studied in mid-lactating dairy cows during LPS-induced inflammation, and there was no evidence of direct improvement of immune functionality.

## 1 Introduction

The annual milk yield of German dairy herds has increased in recent years (1), resulting in a higher susceptibility to infectious diseases and associated negative effects on cow health (2). Dairy cows are exposed to various pathogens, including Gram-negative bacteria, throughout their lives. Therefore, it is crucial to support the immune system of cows to keep them healthy and reduce productivity losses.

Lipopolysaccharides (LPS) are components of the cell wall of Gram-negative bacteria that act as endotoxins, and are known to stimulate an acute phase response (APR) in mammals (3). Because of this characteristics, LPS administration is often used in research as a model to study various aspects of the APR, including the release of pro-inflammatory cytokines, changes in white blood count, and shifts in immune cell activity (4–6). Phagocytosis and the production of reactive oxygen species (ROS) are energy-consuming functions of immune cells that are critical for the innate immune response and are therefore particularly important during APR (7).

L-carnitine plays a crucial role in the energy metabolism of cells by acting as a molecular shuttle, facilitating the transport of free long-chain fatty acids mobilized from adipose tissue across the inner mitochondrial membrane (8). This transport allows fatty acids to be oxidized in the mitochondrial matrix, generating energy in the form of adenosine triphosphate (ATP) (9).

Previous studies provide evidence that L-carnitine may have beneficial effects on LPS-induced sepsis in rats by reducing serum levels of inflammatory cytokines, disease severity, and lethality (10, 11). During an LPS-induced APR in dairy cows, dietary L-carnitine supplementation led to increased plasma carnitine concentrations and was shown to have no significant effect on the progression of clinical signs, except for reduced rumen motility, but resulted in higher insulin concentrations and lower NEFA levels, indicating an effect on lipid metabolism (12). The present study aimed at completing the data of the comprehensive experiment (12) and particularly to fill the knowledge gap regarding the direct influence of dietary L-carnitine supplementation on cellular level of leukocytes and on corresponding leukocyte-derived variables in the blood during a standardized immune challenge.

On the one hand, our aim was to describe the APR in detail in mid-lactating pluriparous dairy cows, when a stable energy status is expected after the transition period. On the other hand, we wanted to elucidate the effects of dietary L-carnitine supplementation on immune and metabolic related variables during an ATP-demanding immune response. We hypothesized that dietary L-carnitine supplementation would modify cellular function of leukocytes during an LPS-induced APR and thus improve immune functionality.

## 2 Materials and methods

### 2.1 Experimental design

The experiment was conducted at the experimental station of the Institute of Animal Nutrition, Friedrich-Loeffler-Institute (FLI), in Braunschweig, Germany in accordance with the German Animal Welfare Act and permitted by the Lower Saxony State Office for Consumer Protection and Food Safety (LAVES, Oldenburg, Germany) (AZ33.19-42502-04-16/2378).

This study is part of a trial that started 42 d prior to expected calving and lasted until d 128 *postpartum (pp)*. The experimental design and feeding data have already been published in detail by Meyer et al. (12, 13) and Kononov et al. (14, 15). In brief, the study included 53 pluriparous German Holstein cows divided into an L-carnitine supplemented group (CAR; n = 27) and a control group (CON; n = 26). Both groups were equally distributed according to number of lactation (2.6 ± 0.8), body condition score (BCS, 3.33 ± 0.51) and body weight (BW, 705 kg ± 75 kg). In accordance with recommendations of nutrient and energy supply of the Society of Nutrition Physiology (GfE), the cows were fed a partial mixed ration, consisting of 50% roughage (70% maize silage and 30% grass silage) provided in feed-weigh troughs (Roughage Intake Control System Insentec B.V., Marknesse, The Netherlands) and 50% concentrate feed, provided in electronic concentrate feeding stations (Insentec B.V., Marknesse, The Netherlands). The CAR-group received 125 g per cow and day of a rumen-protected L-carnitine product (Carneon 20 Rumin-Pro, Kaesler Nutrition GmbH, Cuxhaven, Germany) with the concentrate feed, while the CON-group received a compensatory product with a similar fat content (BergaFat F-100 HP, Berg + Schmidt GmbH & Co. KG, Hamburg, Germany). The amount of the first-mentioned product corresponded to a total L-carnitine intake of 25 g per cow and day. Water was provided *ad libitum*. The study focused on the period between d 110 *pp* and d 128 *pp*, when each cow received a bolus injection of 0.5 µg/kg BW LPS (*E. coli*, Serotype O111:B4, Sigma Aldrich, L2630, St. Louis, Missouri, USA) into a *Vena jugularis externa* by needle puncture on d 111 *pp*. For frequent blood sampling one day *ante injectionem (ai)* until the sampling at 12 h *post injectionem (pi)*, cows previously received an indwelling venous catheter (2.4 mm x 200 mm Teflon catheter, Walter Veterinär-Instrumente e.K., Baruth/Mark, Germany) in the contralateral *V. jugularis externa*. A flex extension (Heidelberger Verlängerung 75 cm, Tierärztebedarf J. Lehnecke GmbH, Schortens, Germany) combined with a three-way valve (WDT Pharmazeutische Handelsgesellschaft mbH, Garbsen, Germany) was used during sampling and the catheter was closed with a stylet (Mandrin, Walter Veterinär-Instrumente e.K., Baruth/Mark, Germany) between blood collections. Prior to participation in the LPS-challenge, two cows were excluded from the study due to an unphysiologically low rectal temperature. Another cow died 24 h *pi* due to an acute shock caused by unrecognized inflammation. As a result, 50 cows completed the trial (n_CON_ = 24, n_CAR_ = 26).

### 2.2 Sample collection

Blood was collected into EDTA, serum, and heparin tubes (Sarstedt AG & Co. KG, Nümbrecht, Germany) and heparin syringes (Werfen, Kirchheim, Germany) at the following timepoints: d 110 *pp* (= 1 d *ai*), d 111 *pp* (LPS injection) at 0.5, 1, 2, 3, 4, 6, 9, 12, 24, 48 and 72 h *pi*, d 118 *pp* (= 7 d *pi*), and d 126 *pp* (= 14 d *pi*). Sampling was performed either by needle puncture or by indwelling catheters from 0.5 h until 12 h *pi*, with 20 ml blood discarded and the catheters flushed with 0.9% sterile saline solution after blood collection. For serum preparation, blood samples were incubated for 1 h at room temperature and then centrifuged (1,950 x g, 15 min, Varifuge 3.0, Heraeus, Hanau, Germany) and stored at -80°C prior to further analysis. To obtain erythrocyte lysate, 2 ml of EDTA blood mixed with 10 ml of cold distilled water was centrifuged (10,000 × g, 10 min, 4°C, Rovall RC6+, Thermo Fisher Scientific, Dreieich, Germany). The top layer forming the erythrocyte lysate was stored at -80°C until further analysis. For plasma preparation, EDTA and heparinized blood was centrifuged (1,950 x g, 15 min, Varifuge 3.0, Heraeus, Hanau, Germany) and the obtained plasma was stored at -80°C until further analysis.

### 2.3 Laboratory methods

#### 2.3.1 Hematology

The automated analyzer Celltac-α (MEK 6450, Nihon Kohden, Qinlab Diagnostik, Weichs, Germany) was used to measure hematological parameters in EDTA whole blood directly after sampling. The following parameters were part of the red blood count: erythrocyte count (RBC), hemoglobin concentration (HGB), hematocrit (HCT), mean corpuscular hemoglobin (MCH), mean corpuscular volume (MCV), mean corpuscular hemoglobin concentration (MCHC), and red cell distribution width (RDW). Platelet-associated parameters included: platelet count (PLT), plateletcrit (PCT), mean platelet volume (MPV), and platelet distribution width (PDW). Furthermore, the white blood cell count consisted of: leukocyte count (WBC) and the number and percentage of lymphocytes, monocytes, granulocytes (neutrophils and basophils combined), and eosinophils. Heparinized blood was used for automated blood gas analysis (GEM Premier 4000, Werfen, Kirchheim, Germany) immediately after sampling to determine oxyhemoglobin (oxy-HGB), deoxyhemoglobin (deoxy-HGB), carboxyhemoglobin (carboxy-HGB) and methemoglobin (met-HGB).

The differential blood count was evaluated by light microscopy (Eclipse E200, Nikon Instruments Europe B.V., Amsterdam, The Netherlands). For this purpose, air-dried blood smears were prepared and stained according to Pappenheim. Granulocytes were manually counted and classified according to their morphology and cytochemical staining characteristics into the following four categories: mature segmented neutrophils, immature banded neutrophils, eosinophils and basophils. A minimum of 100 cells per slide were counted.

#### 2.3.2 Interleukin 6

To determine the concentration of interleukin 6 (IL-6), serum was centrifuged at room temperature (2,200 x g, 5 min, Centrifuge 5427 R, Eppendorf SE, Hamburg, Germany). The supernatant was then analyzed in duplicate by sandwich ELISA using the R&D Systems Bovine IL-6 assay (R&D Systems, Minneapolis, USA) following the manufacturer’s protocol. Optical density was measured at 450 nm using a TECAN Infinite M200 plate reader (Tecan Infinite® 200, Tecan Group Ltd., Männedorf, Switzerland).

#### 2.3.3 Antioxidant enzyme activities

Superoxide dismutase (SOD) and glutathione peroxidase (GPx) activities were determined in erythrocyte lysate. SOD activity was determined using the Ransod superoxide dismutase assay (Randox Laboratories, Crumlin, UK) according to the manufacturer’s protocol with volumes adjusted to fit a 96-well format. GPx activity was determined using the Ransel glutathione peroxidase assay (Randox Laboratories, Crumlin, UK), following the manufacturer’s instructions and also adjusted to a 96-well plate. Both assays were analyzed in duplicate using a TECAN Infinite M200 plate reader (Tecan Infinite^®^ 200, Tecan Group Ltd., Männedorf, Switzerland). Absorbance was measured at 340 nm and 37°C at two time points in the linear range of the reaction. For normalization of the assay results, the erythrocyte lysate was analyzed for HGB concentration using an automated analyzer (Celltac-α MEK 6450, Nihon Kohden, Qinlab Diagnostik, Weichs, Germany).

#### 2.3.4 Ferric reducing ability of plasma

To determine the ferric reducing ability of plasma (FRAP), plasma samples were analyzed based on the procedure of Benzie and Strain (16). FRAP was measured in duplicate with volume adjustment and dilution to fit a 96-well format. The reduction of ferric to ferrous ions at low pH by non-enzymatic antioxidants was measured using a plate reader (Tecan Infinite® 200, Tecan Group Ltd., Männedorf, Switzerland) by determining the changes in absorbance at 593 nm and the formation of blue colored ferrous-tripyridyltriazine complexes after incubation at 37°C for 15 min. Calculations were performed using a calibration curve of Fe^2+^.

#### 2.3.5 Derivatives of reactive oxygen metabolites

The concentration of derivates of reactive oxygen metabolites (dROM) was measured in triplicates using a colorimetric assay based on the method of Regenhard et al. (17). Heparin plasma was incubated with chromogen (0.37 M N,N-Diethyl-1,4phenylendiammomiumsulphate, Merck, Darmstadt, Germany), FeSO_4_ (6 mM, VWR International, Radnor, PA, USA) and acetate buffer (0.01 M, pH 5.0, AppliChem, Darmstadt, Germany) for 120 min at 37°C. A standard curve was generated using standard H_2_O_2_ (Carl Roth, Karlsruhe, Germany). After a 10 min incubation in an ice bath, absorbance was measured at 505 nm using a plate reader (Tecan Infinite® 200, Tecan Group Ltd., Männedorf, Switzerland).

#### 2.3.6 Phagocytosis

Phagocytic activity of peripheral blood mononuclear cells (PBMC) and polymorphonuclear neutrophils (PMN) was measured immediately after sampling by flow cytometry in heparinized blood using the reagent kit PHAGOTEST™ (Glycotope Biotechnology, Heidelberg, Germany). Uptake of fluorescein-labeled opsonized *E. coli* bacteria after 10 min incubation at 37°C served as a phagocytosis marker. In parallel, a negative control was performed by incubating cells with bacteria for 10 min on ice. At least 10,000 cells per sample were analyzed in duplicates and classified into PMN and PBMC based on size and granularity by flow cytometry (FACSCanto™II, BD Biosciences, San Jose, USA, **Supplementary Figures 1A, D**). Data were expressed as percentages and converted to absolute numbers of phagocytosing PMN and PBMC using the results of automated cell counting. The phagocytic capacity of each cell was determined and expressed as mean fluorescence intensity (MFI).

#### 2.3.7 Reactive oxygen species production

The production of intracellular ROS by PMN and PBMC was investigated by flow cytometry (FACSCanto™II, BD Biosciences, San Jose, USA). The method involved intracellular oxidation of non-fluorescent dye dihydrorhodamine-(DHR)-123 to the fluorescent rhodamine-123 (R123) caused by free radicals and hydrogen peroxide. Two assays were prepared for each sample: one unstimulated to determine basal ROS production and one 12-O-tetradecanoylphorbol-13-acetate (TPA) stimulated to induce an oxidative burst by NADPH-oxidase activity. EDTA whole blood was incubated with 40 µM DHR solution and with or without 30 nM TPA for 15 min at 37°C. The erythrocytes were then lysed with lysis buffer (BD Pharm Lyse™, BD Bioscience, San Jose, USA) for 10 min followed by centrifugation (250 x g, 5 min, 4°C, Centrifuge 5427 R, Eppendorf SE, Hamburg, Germany) and resuspension of the cells in Hepes-buffered saline (HBS, 14 mM Hepes, 0.9% NaCl). Flow cytometry (FACSCanto™II, BD Biosciences, San Jose, USA) was used to analyze at least 10,000 cells per sample in duplicates which were categorized by size and granularity into PMN and PBMC (**Supplementary Figures 1A, E**). Data were expressed as percentages and converted to absolute numbers of PMN or PBMC expressing rhodamine 123 fluorescence (R123+) by using the results of automated cell counting. In addition, the capacity of each cell to produce ROS, expressed as MFI, was determined.

#### 2.3.8 Phenotyping of leukocyte subsets

Immunophenotyping was performed within the PBMC population in EDTA whole blood using specific mouse anti-bovine monoclonal antibodies (mAb, all purchased from AbD serotec, Bio-Rad laboratories GmbH, Feldkirchen, Germany). The following phenotypes were determined: T-helper cells (CD4^+^), cytotoxic T-cells (CD8^+^), activated T-cells (CD4^+^CD25^+^), memory T-cells (CD45_Ro_^+^), B-cells (CD21^+^) and monocytes (CD14^+^). A dual staining was performed with CD4: FITC (CC8, IgG2a) and CD8a: RPE (CC63, IgG2a) and another with CD21: PE (CC51, IgG2b) and CD14: FITC (CC-G33, IgG1). To define activated T-cells, a dual staining of CD4: FITC (CC8, IgG2a) and CD25: RPE (IL-A111, IgG1) was performed. A triple staining of CD4: FITC (CC8, IgG2a), CD8: Alexa Fluor^®^ 647 (CC63, IgG2a), and CD45_Ro_: RPE (IL-A116, IgG3) was used to quantify memory T-cells. Negative staining controls were performed with appropriate isotype control antibodies (AbD serotec, Bio-Rad laboratories GmbH, Feldkirchen, Germany). After incubation with the appropriate antibodies for 30 min, erythrocytes were lysed with lysis buffer (BD FACS™ Lysing Solution, BD Biosciences, San Jose, USA) for 10 min on a shaker, followed by centrifugation (250 x g, 5 min, 4°C, Centrifuge 5427 R, Eppendorf SE, Hamburg, Germany) and washing with HBS. Flow cytometry (FACSCanto™II, BD Biosciences, San Jose, USA) was used to analyze at least 10,000 PBMC per sample, and their population was defined by size and granularity (**Supplementary Figures 1A–C**). In addition, the spillover of the selected fluorochromes (FITC, PE, and Alexa Fluor^®^ 647) was balanced using BD FACS Diva software (BD Biosciences, San Jose, USA). Continuous controls were performed using CS&T research beads (BD Biosciences, San Jose, USA) to ensure the quality of the results. Data collected for each PBMC subset were given as percentages as well as converted absolute numbers, using the results of automated cell counting. In addition, the expression density of specific surface markers (CD) on each cell was indicated as MFI.

#### 2.3.9 Clinical chemistry

Serum concentrations of the following parameters were determined photometrically using the automated analyzer Indiko™ Plus (Thermo Fisher Scientific GmbH, Waltham, USA): albumin, cholesterol, glutamate dehydrogenase (GLDH), total protein, urea, alkaline phosphatase (ALP), and alanine aminotransferase (ALT). In addition, aspartate aminotransferase (AST), γ-glutamyltransferase (γ-GT), bilirubin and creatinine values were measured using the clinical chemistry analyzer Eurolyser^®^ CCA (Eurolyser Diagnostica GmbH, Salzburg, Austria).

### 2.4 Calculations and statistics

All parameters were analyzed using the MIXED procedure of SAS version 9.4 (SAS Institute Inc., Cary, NC, USA) with the restricted maximum likelihood method. Group (CON or CAR), time (relative to LPS injection), and the interaction between group and time were included in the model as fixed factors. The appropriate covariance structure (compound symmetry, autoregressive, or unstructured) was selected for each parameter considering the lowest Akaike information criterion (AICC). All following values are presented as least square (LS) means of the group by time interaction with additional standard errors. Statistical effects were declared as significant at p < 0.05. In addition, LS mean comparisons were performed using Tukey’s t-test. The data of eosinophils [%; 10^3^/µl] and monocytes [10^3^/µl], measured by the automated analyzer Celltac-α (MEK 6450, Nihon Kohden, Qinlab Diagnostik, Weichs, Germany), were transformed by adding 10,000 before analysis. A stimulation index (SI) was calculated for the ROS production of PMN and PBMC, by taking the ratio of TPA-stimulated to non-stimulated cells and MFI. Additionally, the ratio of CD4^+^ PBMC and CD8^+^ PBMC was calculated. Pearson’s correlation was calculated using R software (version 4.2.2).

## 3 Results

### 3.1 Erythrogram

**Figure 1:**
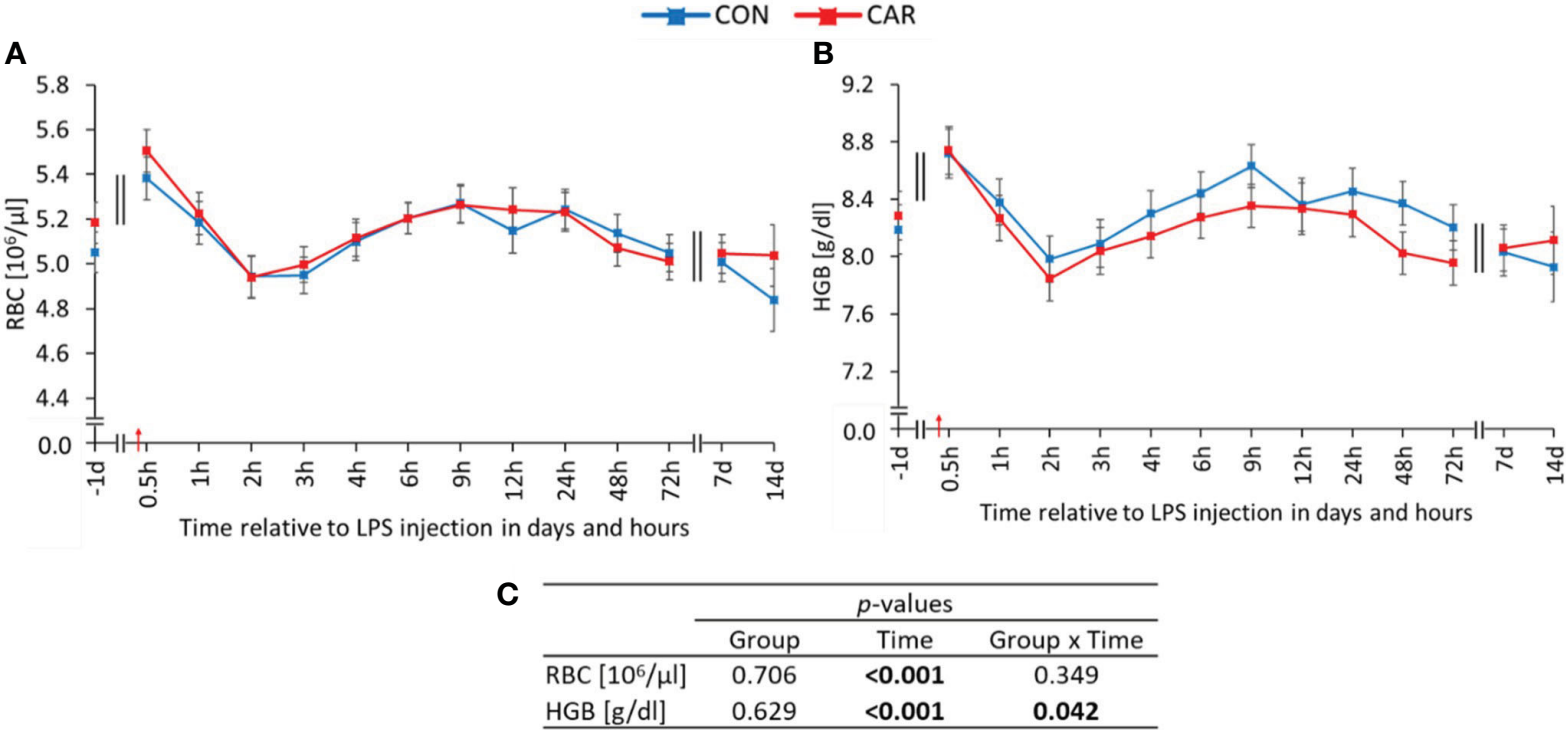
Effects of dietary L-carnitine supplementation (control group = CON; carnitine group = CAR) from 1 day before until 14 days after intravenous LPS injection (red arrow) on red blood count of dairy cows. **(A)** total erythrocyte count (RBC), **(B)** hemoglobin concentration (HGB) measured with an automated cell analyzer. **(C)** Data statistics. Data are shown as least square means ± standard errors.

All parameters of the red blood count except for MCH (16.09 ± 0.02 pg) varied significantly with time (p_T_ ≤ 0.001). Initially, RBC (**Figure 1A**) showed a significant increase from day 1 *ai* to the peak at 0.5 h *pi*, followed by a decrease until 2 h *pi*. Thereafter, a steady increase until 9 h *pi* and a subsequent decrease to pre-challenge counts was observed. HGB (**Figure 1B**) followed a similar course to RBC, but was affected differently by L-carnitine supplementation over time (p_G*T_ = 0.042). Initially, HGB increased to a maximum at 0.5 h *pi*, but only CON peaked significantly, before both groups decreased until 2 h *pi*. Subsequently, CON rose significantly to a second peak at 9 h *pi* and returned to baseline values, whereas CAR remained unchanged until the end of the study. Data statistics are shown in **Figure 1C**. The time course of HCT (**Supplementary Table 1**) was identical to that of RBC. Regarding the erythrocyte indices, MCV (**Supplementary Table 1**) initially reached a significantly higher level for 3 h after LPS administration, followed by a return to baseline until d 14 *pi*, whereas MCHC (**Supplementary Table 1**) did not show significant deviations from baseline throughout the study. RDW (**Supplementary Table 1**) remained at the initial level for 1 week and reached its maximum on the last day of the trial.

### 3.2 Platelets

A significant time-dependent variation (p_T_ ≤ 0.012) was observed for all platelet-associated variables without an effect of dietary L-carnitine supplementation. Both PLT (**Supplementary Table 1**) and PCT (**Supplementary Table 1**) decreased after LPS administration, reaching their nadir at 6 h and 12 h, respectively and returned thereafter to pre-challenge levels. Neither MPV (**Supplementary Table 1**) nor PDW (**Supplementary Table 1**) were significantly different from baseline throughout the study.

### 3.3 Hemoglobin derivatives

Oxy-HGB (65.14 ± 0.321%), Met-HGB (2.86 ± 0.022%) and Deoxy-HGB (31.69 ± 0.311%) (**Supplementary Figure 2B**) were not significantly affected by group, time or their interaction. Only Carboxy-HGB (**Supplementary Figure 2A**) was differently influenced by L-carnitine supplementation over time (p_G*T_ = 0.041). However, the levels of Carboxy-HGB varied inconsistently, resulting in a significant interaction.

### 3.4 Indicators for the oxidative and antioxidative status

Irrespective of the treatment groups, there was a time-dependent variation (p_T_ < 0.001) of all parameters corresponding to the oxidative and antioxidative status. GPx activity (**Supplementary Figure 3A**) maintained at the baseline level until 1 week after LPS administration and ended the trial with the maximum value, whereas SOD activity (**Supplementary Figure 3B**) remained stable throughout the experiment. FRAP (**Supplementary Figure 3C**) started with a 51% decrease to the nadir at 3 h *pi*, before gradually increasing to the pre-challenge level. In contrast to the other parameters, dROM (**Supplementary Figure 3D**) was only measured up to 72 h *pi*. From the beginning of the experiment, it decreased significantly by 46% until 24 h *pi* and returned to the initial level at 72 h after LPS administration.

### 3.5 Leukogram

**Figure 2:**
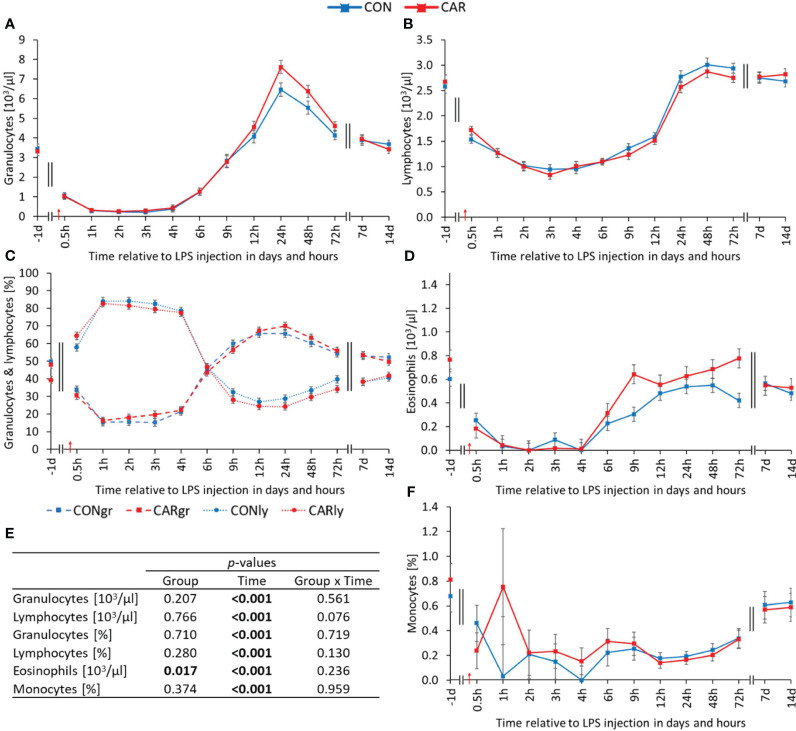
Effects of dietary L-carnitine supplementation (control group = CON; carnitine group = CAR) from 1 day before until 14 days after intravenous LPS injection (red arrow) on white blood count of dairy cows. **(A)** granulocyte count, **(B)** lymphocyte count, **(C)** percentage of granulocytes and lymphocytes, **(D)** eosinophil count, **(F)** percentage of monocytes measured with an automated cell analyzer. **(E)** Data statistics. Data are shown as least square means ± standard errors. CONgr, control group, granulocytes; CARgr, carnitine group, granulocytes; CONly, control group, lymphocytes; CARly, carnitine group, lymphocytes.

Massive kinetic changes were observed in the leukogram. All parameters of the white blood cell count varied significantly with time (p_T_ < 0.001) and were therefore influenced by LPS administration. The total leukocyte count has already been published by Meyer et al. (12) and showed a biphasic response to the immune challenge. Immediately after LPS injection, there was an 83% decrease to a minimum at 3 h *pi*, followed by a significant increase to a peak at 24 h *pi* and a return to baseline level until the end of the trial. Similar changes over time were observed for the granulocyte count (**Figure 2A**). Immediately after LPS injection, it decreased by 93% to a minimum at 2 h *pi*, after which it increased to more than double of the initial value at 24 h *pi*. Thereafter, granulocytes returned to baseline level until d 14 *pi*. Regarding the absolute lymphocyte values (**Figure 2B**), there was a 66% decrease until 3 h *pi*, followed by a significant increase until 24 h *pi* to the initial level, which was maintained until the end of the experiment. In comparison, the percentages (**Figure 2C**) of the two aforementioned cell populations had opposite biphasic courses. While the percentage of granulocytes decreased significantly below the initial level until 4 h *pi*, the percentage of lymphocytes increased to a significantly higher level during the same period. Subsequently, the proportions of granulocytes and lymphocytes reached their maximum and minimum, respectively, before both returned to initial values. The mean eosinophil count (**Figure 2D**) was on average 25% higher in CAR than in CON (p_G_ = 0.017) over time. Both groups initially dropped to a significantly lower level after LPS administration, which lasted for 6 h, before returning to baseline at 9 h *pi* and remaining at this level until 14 d *pi*. Data statistics are shown in **Figure 2E**. The percentage of eosinophils (**Supplementary Table 2**) differed in the same way as the total eosinophils, but was not significantly influenced by L-carnitine. During the first 4 h after LPS administration, the percentage of monocytes (**Figure 2F**) decreased by 90% with a large animal-specific variation to the mean nadir, followed by a slight increase until the end of the study. The absolute number of monocytes (**Supplementary Table 2**) dropped to one fifth of the initial level immediately after LPS administration and remained at this level for 3 days, before recovering until 7 d *pi*.

**Figure 3:**
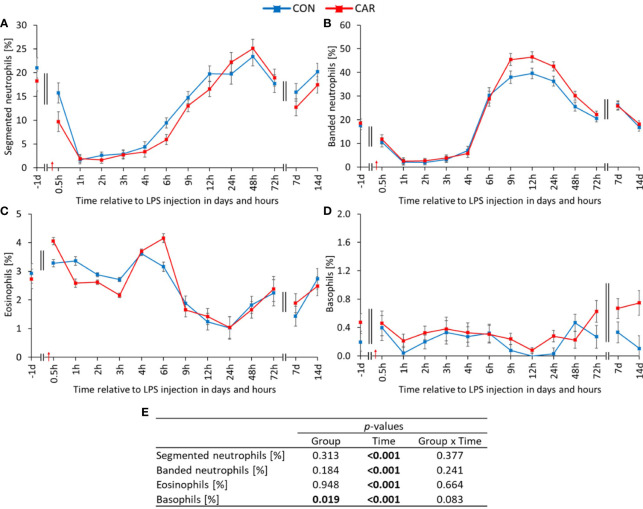
Effects of dietary L-carnitine supplementation (control group = CON; carnitine group = CAR) from 1 day before until 14 days after intravenous LPS injection (red arrow) on differential blood count of dairy cows. **(A)** segmented neutrophils, **(B)** banded neutrophils, **(C)** eosinophils and **(D)** basophils counted manually on stained blood smears. **(E)** Data statistics. Data are shown as least square means ± standard errors.

Each manually counted white blood cell population fluctuated significantly over time (p_T_ < 0.001). The proportion of segmented neutrophils (**Figure 3A**), representing the mature granulocytes, showed a biphasic course. Immediately after LPS administration, it decreased rapidly to a minimum at 1 h *pi* and returned to the pre-challenge level at 12 h *pi*. One week after the challenge, the mature granulocytes decreased significantly for a second time, before recovering until the end of the study. Consistent with the above, the percentage of banded immature neutrophils (**Figure 3B**) decreased sharply with LPS injection, but then exceeded the baseline value more than twofold at 12 h *pi*. Thereafter, banded neutrophils decreased continuously, except for a second peak at 7 d *pi*. The percentage of eosinophils (**Figure 3C**) remained at the initial level until 9 h *pi* and then decreased until 24 h *pi*. From 48 h *pi* until the end of the study, the baseline level was reached again. Notably, the percentage of basophils (**Figure 3D**) showed a significant group effect (p_G_ = 0.019) in addition to the time effect. On average, CAR was 77% higher than CON. Data statistics are shown in **Figure 3E**. The manually counted lymphocyte percentage (**Supplementary Table 2**) was also affected by LPS administration. It immediately increased to a higher level until 4 h *pi* and then decreased to a minimum at 12 h *pi*, before slowly recovering. The percentage of monocytes (**Supplementary Table 2**) decreased to a significantly lower level for 6 h after the challenge and returned to baseline level from 9 h *pi*.

### 3.6 Interleukin 6

**Figure 4:**
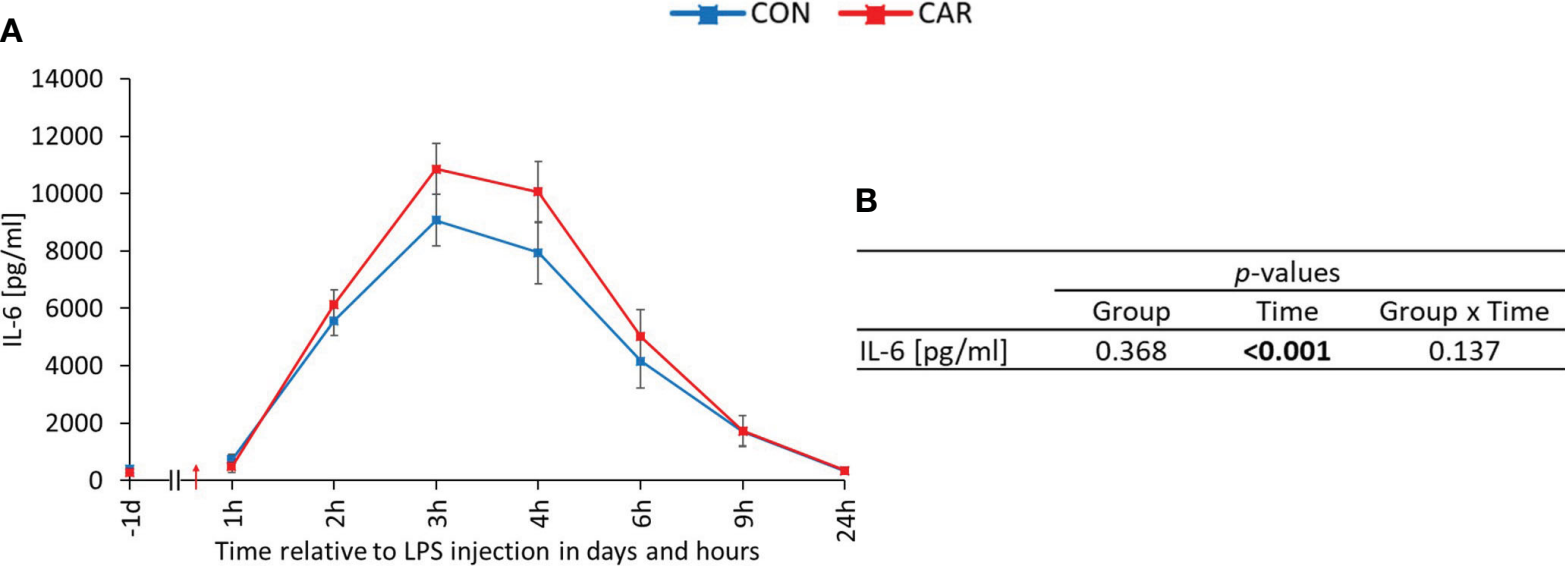
Effects of dietary L-carnitine supplementation (control group = CON; carnitine group = CAR) from 1 day before until 24 hours after intravenous LPS injection (red arrow) on interleukin 6 (IL-6) of dairy cows. **(A)** interleukin 6 concentration measured with ELISA. **(B)** Data statistics. Data are shown as least square means ± standard errors.

Independently of diet, IL-6 (**Figure 4A**) was significantly affected by LPS injection (p_T_ < 0.001), but in contrast to the other parameters, it was only measured up to 24 h *pi*. From the beginning of the experiment until 3 h *pi*, it significantly increased 30-fold and returned to the initial level at the end of the measurement period. Data statistics are shown in **Figure 4B**.

### 3.7 Functional properties of leukocytes

**Figure 5:**
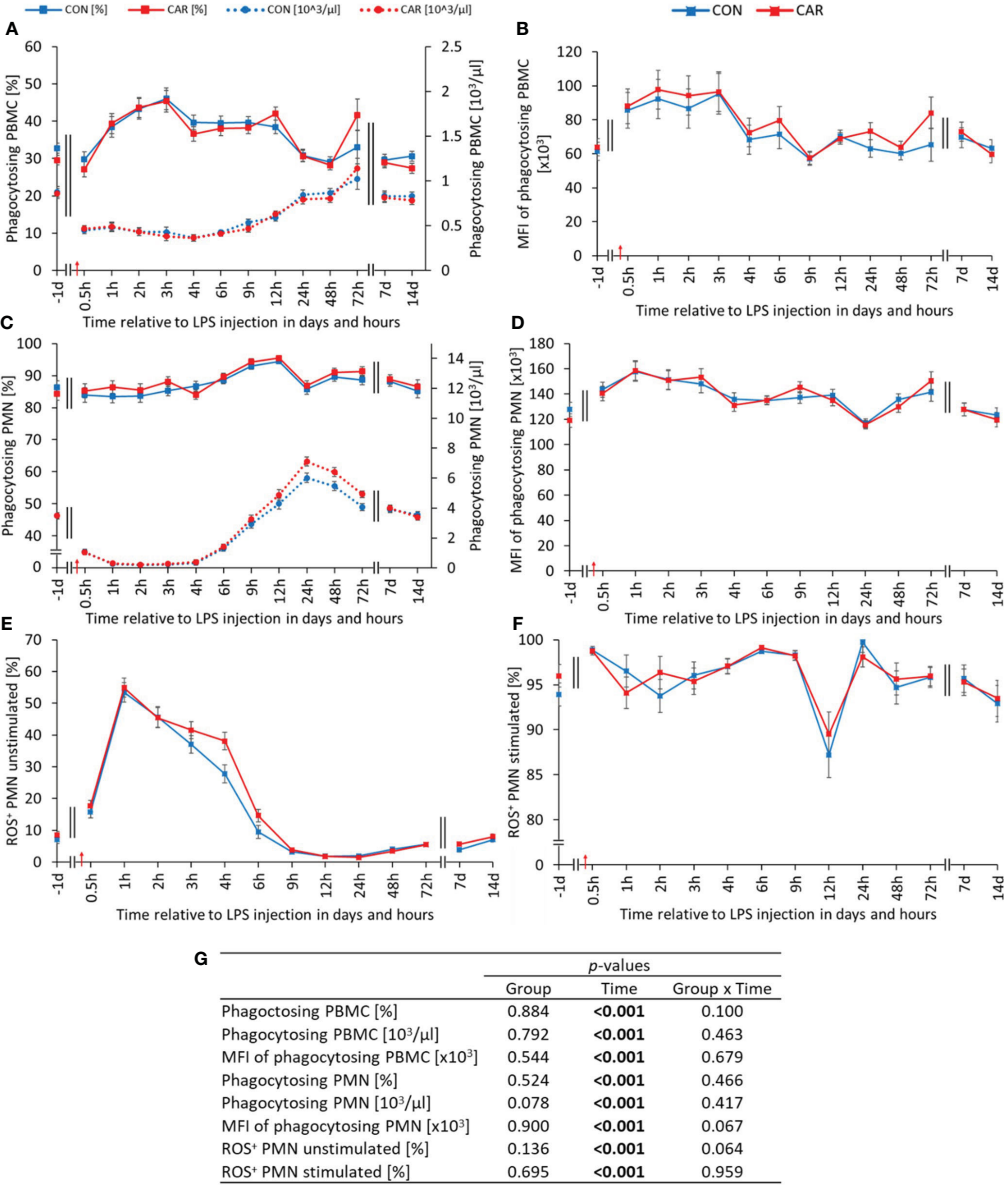
Effects of dietary L-carnitine supplementation (control group = CON; carnitine group = CAR) from 1 day before until 14 days after intravenous LPS injection (red arrow) on functional properties of leukocytes of dairy cows. **(A)** percentage and calculated number of phagocytosing peripheral blood mononuclear cells (PBMC), **(B)** mean fluorescence intensity (MFI) of phagocytosing PBMC, **(C)** percentage and calculated number of phagocytosing polymorphonuclear neutrophils (PMN), **(D)** MFI of phagocytosing PMN, **(E)** percentage of unstimulated reactive oxygen species (ROS+) producing PMN, **(F)** percentage of 12-O-tetradecanoylphorbol-13-acetate (TPA)-stimulated ROS+ PMN determined by flow cytometry. **(G)** Data statistics. Data are shown as least square means ± standard errors.

All measured functional parameters of leukocytes, consisting of phagocytosis and ROS production, varied significantly over time (p_T_ < 0.001). The proportion of phagocytosing PBMC (**Figure 5A**) increased by 47% in both groups after LPS administration until 3 h *pi* and then decreased to reach the baseline level at 24 h *pi*. In contrast, absolute phagocytosing PBMC (**Figure 5A**) decreased to a significantly lower level after the challenge and recovered continuously from 9 h *pi* until the end of the study. Looking more closely at the capacity of phagocytosing PBMC (**Figure 5B**), expressed as MFI, the course of their graph is similar to its percentage with a peak at 3 h *pi*, but with the difference that the baseline level was already reached at 4 h *pi*. Irrespective of L-carnitine supplementation, the proportion of phagocytosing PMN (**Figure 5C**) maintained the initial level for 6 h before peaking significantly at 12 h *pi* and decreasing to the pre-challenge level until the end of the study. Absolute phagocytosing PMN (**Figure 5C**) differed in the same way as granulocyte counts and showed a biphasic course with a minimum at 2 h *pi* and a 32-fold higher maximum at 24 h *pi*. A time-dependent variation was also observed for the MFI of phagocytosing PMN (**Figure 5D**), which rose with LPS-administration to a maximum at 1 h *pi* and returned to the prechallenge level from 12 h *pi* for the rest of the experiment, except for another peak at 72 h *pi*.

LPS administration had an extensive impact on the basal ROS-production of PMN (**Figure 5E**). Immediately after challenge, there was a steep increase until 1 h *pi*, followed by a 97% decrease until 24 h *pi* and a subsequent recovery until 14 d *pi*. However, the calculated absolute ROS-producing PMN showed a contrasting course, as shown in **Supplementary Table 3**. The ROS-formation per cell, expressed as MFI of unstimulated PMN (**Supplementary Table 3**), varied inconsistently with time. The peak occurred at 3 h *pi*, followed by a continuous decrease until 9 h *pi* and a subsequent return to the initial level. The proportion of TPA-stimulated ROS-forming PMN (**Figure 5F**) fluctuated nonspecifically over time until 9 h *pi* and dropped rapidly to a nadir at 12 h *pi*, when only 88% of PMN could be stimulated, before maintaining the baseline level until the end of the trial. Data statistics are shown in **Figure 5G**. In comparison, the time course of the calculated absolute counts of the latter (**Supplementary Table 3**) was similar to that of the absolute granulocyte counts. The corresponding changes in the MFI of stimulated PMN (**Supplementary Table 3**) varied slightly with time. The additionally calculated stimulation index (SI) of ROS-producing PMN (**Supplementary Table 3**) showed a biphasic time course with a minimum at 1 h *pi* and a maximum at 12 h *pi*. At the end of the experiment, it returned to the initial level after a decrease of 81%. The SI of MFI of ROS-producing PMN (**Supplementary Table 3**) remained at the same level for 4 h after the immune challenge and showed the significant highest capacity at 9 h *pi*, followed by a reduction to baseline values until the end of the trial. Related parameters of ROS-formation in PBMC were not significantly affected by L-carnitine supplementation, but were strongly influenced by LPS administration, as shown in **Supplementary Figure 4**.

### 3.8 Phenotyping of leukocyte subsets

**Figure 6:**
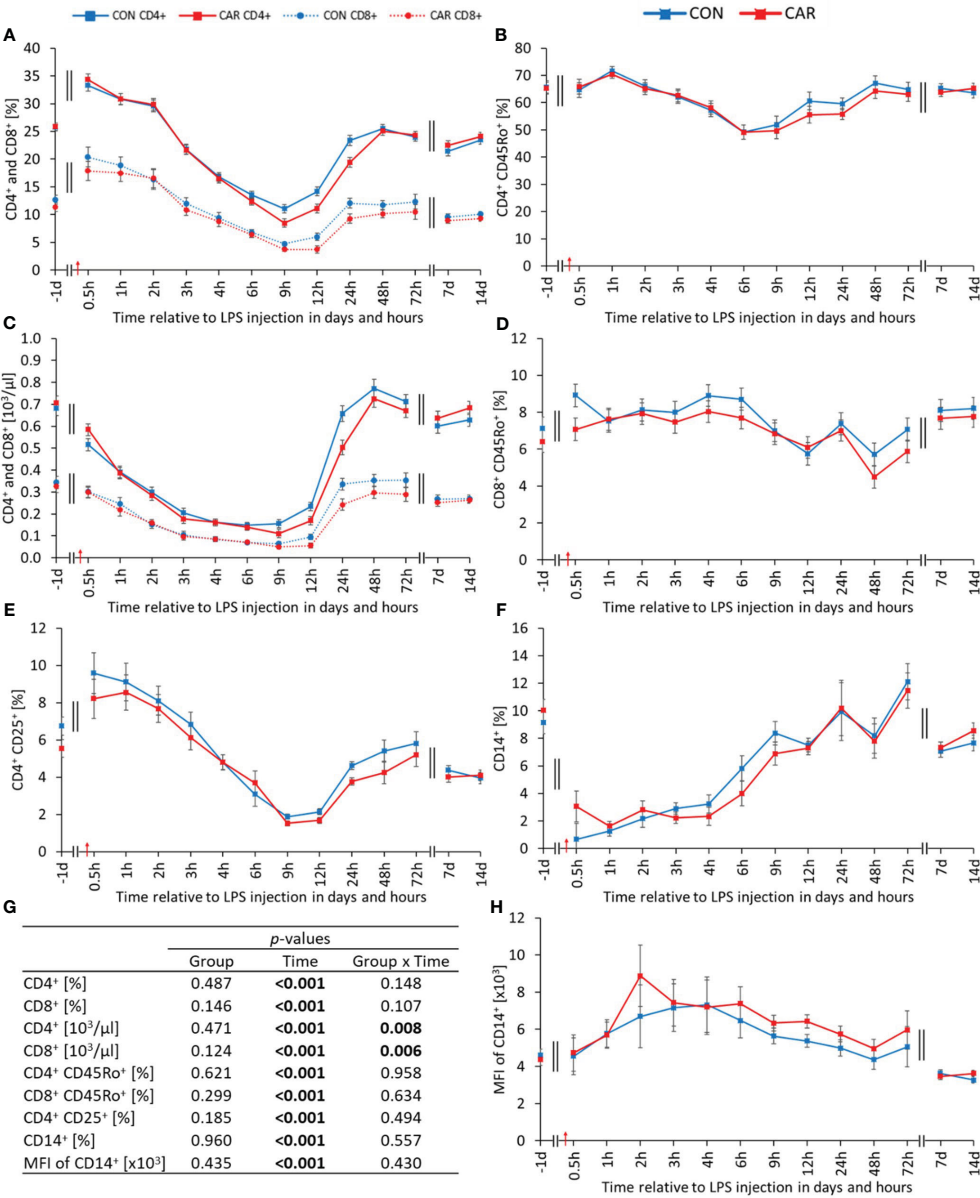
Effects of dietary L-carnitine supplementation (control group = CON; carnitine group = CAR) from 1 day before until 14 days after intravenous LPS injection (red arrow) on phenotypes of leukocyte subsets of dairy cows. **(A)** percentage of T-helper (CD4^+^) and cytotoxic T (CD8^+^) cells, **(B)** percentage of memory T-helper cells (CD4^+^CD45Ro^+^), **(C)** calculated absolute number of CD4^+^ and CD8^+^, **(D)** percentage of memory cytotoxic T-cells (CD8^+^CD45Ro^+^), **(E)** percentage of activated T-cells (CD4^+^CD25^+^), **(F)** percentage of monocytes (CD14^+^), **(H)** mean fluorescence intensity (MFI) of CD14^+^ measured by flow cytometry. **(G)** Data statistics. Data are shown as least square means ± standard errors.

All determined leukocyte phenotypes and associated parameters changed significantly with time (p_T_ < 0.001). The percentage of T-helper (CD4^+^) (**Figure 6A**) and cytotoxic T (CD8^+^) cells (**Figure 6A**) relative to total PBMC, showed statistically similar courses. Both rose immediately after LPS administration to the maximum at 0.5 h *pi* and dropped afterwards significantly to a nadir at 9 h *pi*. The proportion of CD4^+^ returned to the level before LPS administration within 48 h *pi* and reached a significantly lower level compared to the baseline at 72 h *pi*, which was maintained until the end of the study. In comparison, the proportion of CD8^+^ reached the initial level within 24 h *pi*, remained there until 72 h *pi*, and reached a significantly lower level compared to the baseline at 7 d *pi*, which was maintained until the end of the trial. Absolute numbers of T-helper cells (CD4^+^) (**Figure 6C**) were differently affected by L-carnitine supplementation over time (p_G*T_ = 0.008). The two groups decreased significantly after LPS injection, but CON reached its minimum after 6 h *pi* and CAR after 9 h. Both groups returned to their initial values, with CON reaching this level after 24 h *pi* and CAR after 48 h *pi*. Absolute cytotoxic T-cells (CD8^+^) (**Figure 6C**) were also significantly affected by the interaction of group and time (p_G*T_ = 0.006). CON and CAR decreased at the beginning to a significant minimum at 9 h *pi* and reached the baseline from 24 h *pi* until the end of the experiment. Throughout the study, cytotoxic T-cells of both groups followed opposite directions several times, resulting in a significant interaction. The percentage of memory T-helper cells (CD4^+^CD45_Ro_^+^) (**Figure 6B**) relative to total CD4^+^ increased to a peak at 1 h *pi*, followed by a significant decrease to a nadir at 6 h *pi*. It then rose again and remained at the pre-challenge level from 48 h *pi* until the end of the study. The proportion of memory cytotoxic T-cells (CD8^+^CD45_Ro_^+^) (**Figure 6D**) relative to total CD8^+^ rose to a maximum until 4 h *pi*, before slightly decreasing to a minimum at 48 h *pi* and returning to the initial level at 72 h *pi*. The percentage of activated T-cells (CD4^+^CD25^+^) (**Figure 6E**) relative to total CD4^+^ increased slightly until 0.5 h and then decreased sharply by 81% to a nadir at 9 h *pi*. From 24 h *pi* until the end of the trial, a constant but significantly lower level than the initial one was maintained. The additionally determined MFI as well as the ratio of CD4^+^ and CD8^+^ varied significantly over time, with slight changes after LPS injection, as shown in **Supplementary Table 4**. Regardless of L-carnitine supplementation, the percentage of monocytes (CD14^+^) (**Figure 6F**) relative to total PBMC, dropped by 85% after the immune challenge to a minimum at 1 h *pi* and recovered continuously until the end of the study, reaching a significant peak at 72 h *pi*. Data statistics are shown in **Figure 6G**. In comparison, the MFI of the latter (**Figure 6H**) showed a slight increase until 2 h *pi*, followed by a decrease, resulting in significantly lower values than at the beginning of the experiment. The time course of the proportion of B-cells (CD21^+^) (**Supplementary Table 4**) relative to the total PBMC varied in a biphasic manner. This cell population peaked at 3 h *pi* and reached a minimum at 12 h *pi*. For the rest of the study, it remained at a significantly lower level, compared to the initial one. The corresponding MFI (**Supplementary Table 4**) declined to a nadir at 4 h *pi* and returned to prechallenge values at 72 h *pi*. Furthermore, the calculated absolute numbers of CD14^+^ and CD21^+^ (**Supplementary Table 4**) fluctuated with time, with pronounced changes until 24 h after LPS administration.

### 3.9 Clinical chemistry

**Figure 7:**
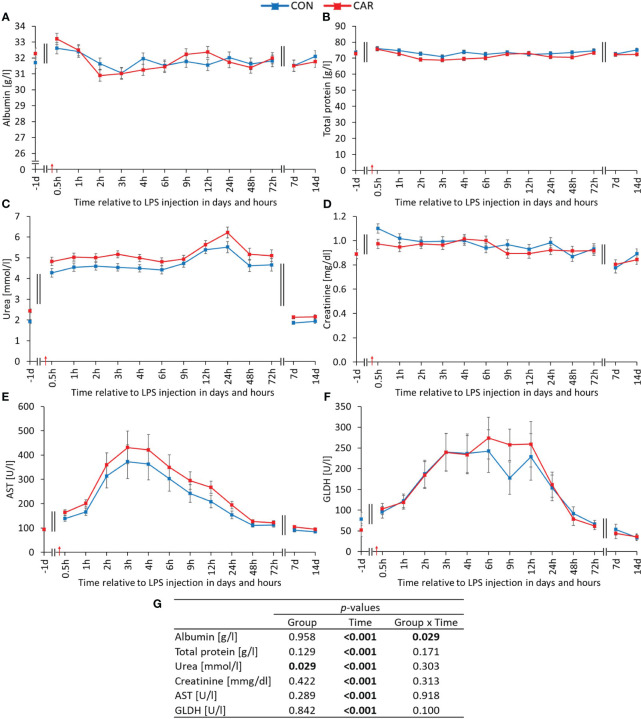
Effects of dietary L-carnitine supplementation (control group = CON; carnitine group = CAR) from 1 day before until 14 days after intravenous LPS injection (red arrow) on clinical chemistry parameters of dairy cows. **(A)** albumin, **(B)** total protein, **(C)** urea, **(D)** creatinine, **(E)** aspartate aminotransferase (AST), **(F)** glutamate dehydrogenase (GLDH) measured with automated clinical chemical analyzer. **(G)** Data statistics. Data are shown as least square means ± standard errors.

A significant time-dependent variation (p_T_ ≤ 0.001) was observed for all clinical chemistry parameters. Interestingly, albumin (**Figure 7A**) was differently affected by treatment over time (p_G*T_ = 0.029). The baseline level in CON was kept constant until the end of the study, while in CAR there was a significant decrease by 4% from baseline to the minimum until 2 h *pi*. Subsequently, from 4 h *pi* until the end of the observation period, the initial level in CAR was reached again. With the exception of a significant minimum at 3 h *pi*, total protein (**Figure 7B**) did not vary from the initial value regardless of L-carnitine supplementation. The urea level (**Figure 7C**) in CAR was on average 11% higher than in CON throughout the study (p_G_ = 0.029). After a doubling of the initial value immediately after LPS administration, a significantly higher and further increasing level with a maximum at 24 h *pi* was maintained until 72 h *pi*. Thereafter, a return to baseline was observed from 7 d *pi* until the end of the study. The highest creatinine value (**Figure 7D**) appeared 0.5 h after LPS injection, followed by a slight decrease back to baseline until 6 h *pi*. The AST activity (**Figure 7E**) reached the 4-fold of the initial value at 3 h *pi* and then slowly declined to the pre-challenge level until the end of the trial. A similar 4-fold increase and subsequent decrease was observed for the GLDH activity (**Figure 7F**), but with a maximum value at 6 h *pi*. Data statistics are shown in **Figure 7G**. Taking a closer look at the cholesterol concentration (**Supplementary Table 5**), it maintained the initial level until 72 h *pi*, before reaching a significantly lower level from d 7 *pi* until the end of the study. As shown in **Supplementary Table 5**, all other liver-associated parameters (γ-GT, ALP, ALT, bilirubin) varied significantly over time, with an increase due to LPS administration and a subsequent decrease to baseline until the end of the experiment.

## 4 Discussion

The present experiment was conducted to evaluate the potential benefits of dietary L-carnitine supplementation in mid-lactating dairy cows, with particular emphasis on immune cell function during systemic LPS-induced inflammation. The systemic effects of immune challenge in cattle have been previously described by several authors (18–20), but not to the extent of the present study and under the conditions of L-carnitine supplementation.

The red blood count is likely to be indirectly affected by LPS, as there is no evidence in the literature for a direct interaction of LPS with erythrocytes. Immediately after LPS injection, RBC increased significantly, accompanied by an increase in lactate, a decrease in blood pH, and an increase in respiratory rate, as published by Meyer et al. (12). These responses indicate reduced oxygen availability in parts of the organism due to endotoxemia, which needs to be regulated by several mechanisms. One mechanism could be the release of additional erythrocytes into the bloodstream, since oxygen transport is their main function (21). In some mammals, the spleen is known to act as a storage pool for erythrocytes that can be released in oxygen demanding situations (22, 23). The present data suggest that a similar mechanism may occur in cows immediately after LPS injection to meet their oxygen requirements. Hematocrit is affected by both the number and size of blood cells and the distribution of body fluids. Since erythrocytes make up approximately 99% of the blood cells (24), this cell population has the greatest impact on hematocrit and is closely correlated with it in the present study (r = 0.82). Fluid balance is affected by water intake and fluid loss, such as sweating (24). For technical reasons, water intake could not be recorded during the relevant period as a limitation of this study. Therefore, the immediate increase in hematocrit and erythrocyte count could be related to reduced water intake in addition to a possible direct effect on erythrocyte count. In an early study by Griel et al. (25) in which lactating cows were challenged intravenously with 1 mg LPS in form of a bolus injection and blood samples were taken at high frequency, no significant changes in hematocrit, erythrocyte count, and hemoglobin concentration were observed. Oh et al. (26) infused pluriparous cows intravenously with 1 µg/kg BW LPS and found a higher hemoglobin concentration 8 h after the end of LPS infusion compared to the zero sample, whereas red blood cells were not affected by LPS. Our results showed a significant interaction (Group x Time) for the hemoglobin concentration and carboxy-HGB with a high individual variation. The related parameters of the red blood count as MCHC were not affected by L-carnitine supplementation in the present experiment, and comparable studies are scarce so that the effects cannot be plausibly explained.

In particular, the white blood cell count was massively affected by the LPS injection. The already published blood leukocyte concentration (12) showed a typical biphasic course (4, 25) with leukopenia followed by leukocytosis. In the present study, leukopenia occurred until 9 h after the immune challenge, which is caused, in part, by an uneven distribution of leukocytes towards the marginated pool due to aggregation, margination, and adhesion of monocytes and granulocytes to the endothelium (27). In addition, another important mechanism involved in the inflammatory response is the migration of immune cells into the surrounding tissues (28–30), resulting in a lower number of leukocytes in the bloodstream. The subsequent leukocytosis is thought to be mainly due to the release of granulocytes from bone marrow reserves (31, 32).

The present results for neutrophil and lymphocyte numbers are consistent with a study by Chandler et al. (4) in which cows were intravenously infused with 0.0625 µg/kg BW LPS for 1 h and blood samples were collected up to 72 h after challenge. In addition, the same dynamics of these populations were observed in a study by Kvidera et al. (33) in which cows received a bolus injection of 1.5 µg/kg BW LPS and were sampled up to 12 h *pi*. Although in the present study the numbers of both populations were reduced immediately after LPS, it should be noted that for the first 6 h *pi* there was a shift in their ratio in favor of the lymphocytes and, therefore, a greater disappearance of neutrophils from the bloodstream. To explain the increase in granulocyte count from 2 h until 24 h after LPS injection, it is necessary to take a closer look at the results of the differential blood count. The detection of a sharp increase in banded, and therefore immature, neutrophils up to 12 h *pi* indicates their new formation and release from the bone marrow reserves. There is evidence, that the storage pool, which is thought to represent two-thirds of the bone marrow reserves, consists of both banded and segmented neutrophils (31). This implies that the slightly delayed increase in segmented neutrophils is due to their release from the bone marrow and the development of banded to segmented neutrophils in the blood. The origin of the increase in lymphocytes starting 3 h after the immune challenge cannot be conclusively determined. They might be recirculated from lymphoid tissue, released from reservoirs in secondary lymphatic organs, or newly produced (34).

In order to classify the result of the eosinophil count, it is important to take a look at the data from the first part of the feeding experiment, which included the calving of the cows. Consistent with the group effect in the current part of the study, the number of eosinophils was also higher in CAR during the transition period (15), underlining the influence of L-carnitine on this cell population. Typically, eosinophils are associated with parasite defense (35), but they are also involved in antibacterial immunity by several mechanisms. In addition to phagocytosis, they can secrete bactericidal granule contents after direct activation by the pathogen, either by cytolysis, exocytosis, or piecemeal degranulation (36). Since it has been shown that L-carnitine can improve membrane stability in human erythrocytes (37) and reduce apoptosis in mouse fibroblasts (38) as well as in mouse embryos (39), a similar membrane protective mechanism might occur in eosinophils. Due to the higher availability of L-carnitine, the secretion mechanism of eosinophils in CAR might be membrane-preserving, whereas cell lysis might be more likely to occur in CON, resulting in the group difference.

The manually counted basophil percentage was also higher in CAR than in CON, and an interaction (Group x Time) was already observed in the transition period (15). Since the immune function of basophils is partly similar to that of eosinophils, based on the secretion of granules (40), the theory of a membrane-stabilizing effect of L-carnitine might also be relevant in this cell population. However, the method of manual counting may not be sensitive enough in this case due to the very low percentage of basophils in combination with only 100 cells counted per slide. Therefore, the effect on basophils in the present study must be interpreted with caution.

IL-6 is a well-studied cytokine which plays a central role in initiating and coordinating the APR by influencing the production of acute phase proteins, promoting inflammation, regulating fever, modulating hematopoiesis, influencing metabolism, and contributing to tissue repair and remodeling (41–43). LPS administration induced an APR and consequently IL-6 responded strongly to the immune challenge with an increase to a maximum at 3 h *pi* followed by a decrease to the baseline until 24 h *pi*. A study by Burdick et al. (44) investigated the effect of 0.5 µg/kg LPS injected intravenously into steers and observed a similar time course for IL-6 concentration with the difference of an earlier maximum at 2.5 h pi and an earlier return to baseline at 4 h pi. Since no sample was collected at the 2.5 h time point in our experiment, the different peaks may be due to different frequency of blood sampling. Carroll et al. (45) showed that different breeds of cattle may have different innate immune responses. Although IL-6 levels did not differ significantly between breeds in the aforementioned study, there was a difference in other APR-associated parameters, such as cortisol. Due to the anti-inflammatory properties of cortisol, including the inhibition of IL-6 production (46), a relation between these parameters is very likely and may be supported by a significant correlation (r = 0.58) between IL-6 and cortisol in our study. As the maximum cortisol level in the study by Burdick et al. (44) was about 20% higher than the maximum in the previously published results of our study (12), a faster cortisol-induced down-regulation of APR could have resulted in an earlier reduction of IL-6. In addition, the sex, age, feeding, and housing of the cattle, which varied between studies, may have had an effect on how they coped with endotoxemia, and therefore also IL-6 and cortisol levels.

L-carnitine had no effect on the functional properties of leukocytes, but LPS injection induced fluctuations in all related parameters. First of all, both PMN and PBMC significantly increased their phagocytic capacity as early as 0.5 h *pi*, so that this regulation seems to respond most rapidly to LPS. From 1 h *pi*, the proportion of phagocytosing PBMC also increased significantly, probably due to an increased activity of monocytes as phagocytosing cells. LPS is bound to LPS-binding protein (LBP) which then enables the interaction of LPS with CD14, allowing CD14 to present LPS to Toll-like receptor 4 (TLR4) and initiate immune signaling pathways (47). Among blood cells, monocytes typically have the highest expression of CD14 (48), resulting in an early increase in phagocytic activity by signaling cascades in the present study. It is important to note that bovine monocytes are characterized by the expression of both CD14 and CD16, resulting in three subpopulations: CD14^++^ CD16^−^, CD14^++^ CD16^+^, CD14^−^ CD16^++^ (49). In this study, only CD14-positive monocytes were examined, but it is known that all three subpopulations differ in functionality and responsiveness to LPS (49). This may lead to different changes between these subsets after LPS-injection, which cannot be explained in the present study, due to the lack of combined staining with CD14 and CD16 antibodies. Notably, the percentage of phagocytosing PMN maintained its high initial level for 6 h after LPS injection and started to increase when the percentage of phagocytosing PBMC decreased slightly from its maximum. This suggests a form of counter regulation to maintain high phagocytic activity within the phagocytes. A similar regulatory pattern is seen in the percentage of ROS-producing PMN and PBMC. ROS production seems to be the first response of PMN to invading pathogens such as LPS, as the maximum percentage is already reached at 1 h *pi*. The percentage of ROS-producing PBMC started to increase from 4 h *pi*, while the percentage of ROS-producing PMN was falling. These detected relationships between PMN and PBMC functionality underline the tight regulation of APR at the functional level of immune cells by cytokines and other signaling molecules (50).

Except for the absolute numbers of T-helper (CD4^+^) and cytotoxic T (CD8^+^) cells, L-carnitine had no effect on different PBMC subsets. Our results show a significant interaction (Group x Time) for these phenotypes with transiently reduced levels in CAR. Independently of LPS, Athanassaki et al. showed reduced proportions of CD4^+^ and CD8^+^ lymphocytes in the spleen of mice due to L-carnitine supplementation (51). Furthermore, Tschaikowsky et al. (52) observed in humans that sepsis survivors had lower blood CD4^+^ and CD8^+^ lymphocyte counts than non-survivors, suggesting a beneficial effect on health of lower levels of these cell populations during periods of immune challenge.

Alterations in protein metabolism are known to occur during APR due to the production of acute phase proteins (APP) in the liver (53), resulting in increased protein requirements. The present experiment showed higher milk urea levels (12) as well as higher blood urea levels due to LPS injection. Meyer et al. discussed the catabolism of excess amino acids from muscle breakdown that were not used for APP synthesis as the reason for the increased milk urea level (12). The significant correlation between blood and milk urea (r = 0.70) suggests that the LPS-induced increase has the same physiological origin. Furthermore, significantly higher urea levels were observed in CAR for both milk and blood throughout the study, but the mechanism for this circumstance is not clear. Since L-carnitine is an amino acid derivative, one might assume that an excess of this agent would also lead to degradation via the urea cycle in the liver. This is contradicted by the fact that Carlson et al. (54) found lower plasma urea N levels with increasing dietary L-carnitine in periparturient dairy cows, which was not investigated in the present study. Additionally, the effects of L-carnitine on protein metabolism may depend on the metabolic state of the cow which varies between the calving period and the LPS-induced APR.

The present study revealed that CAR had no advantage or disadvantage over CON on the energetic level of immune cells, as indicated by their response to the LPS-challenge. As already described (12), the control group was also able to cover their L-carnitine requirements and was not in a deficiency situation. Furthermore, it is known that activated lymphocytes and neutrophils tend to meet their energy requirements via glycolysis (55), and beta-oxidation may not be the main metabolic pathway in these cells for generating ATP.

In conclusion, supplementation of 25 g L-carnitine per cow and day has only isolated effects on blood parameters in mid-lactating dairy cows during an LPS-induced inflammation and does not directly improve functional properties and thus the energy metabolism of immune cells.

However, L-carnitine supplementation resulted in higher eosinophil counts and basophil proportion which may be due to an improved membrane stability.

## Data availability statement

The original contributions presented in the study are included in the article/**Supplementary Material**. Further inquiries can be directed to the corresponding author and will be available at Zenodo.org.

## Ethics statement

The animal study was approved by Lower Saxony State Office for Consumer Protection and Food Safety (LAVES, Oldenburg, Germany). The study was conducted in accordance with the local legislation and institutional requirements.

## Author contributions

LS: Data curation, Formal analysis, Visualization, Writing – original draft. JF: Conceptualization, Data curation, Methodology, Supervision, Writing – review & editing. SK: Data curation, Methodology, Writing – review & editing. SB: Data curation, Writing – review & editing. UM: Writing – review & editing. CV: Supervision, Writing – review & editing. KH: Conceptualization, Funding acquisition, Project administration, Writing – review & editing. SD: Conceptualization, Formal analysis, Funding acquisition, Project administration, Supervision, Writing – review & editing.
