# Supplementary material for: Dietary L-carnitine supplementation modifies blood parameters of mid-lactating dairy cows during standardized lipopolysaccharide-induced inflammation
Source: Front Immunol. 2024 May 13;15:1390137. doi: 10.3389/fimmu.2024.1390137 (PMC11130594; doi:10.3389/fimmu.2024.1390137)
Supplement: Supplementary file 1 [file DataSheet_1.docx]

**Supplementary Material**

**
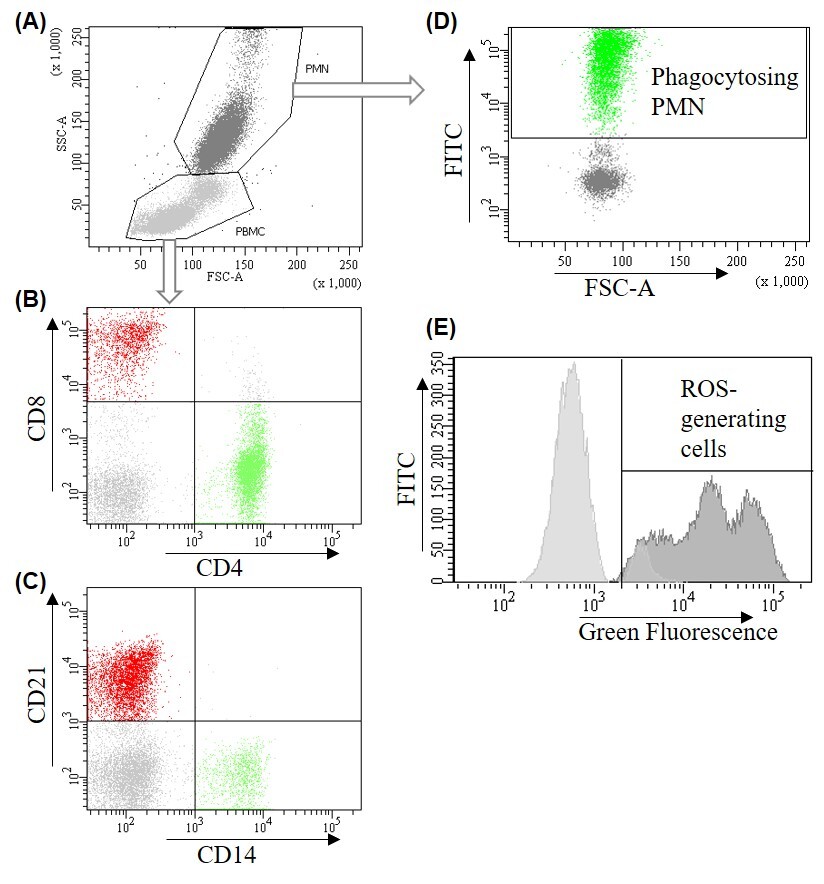
Supplementary Figure 1:**

**Supplementary** **Figure** **1:** Flow cytometry gating strategy showing the quantification of lymphocyte subsets from bovine peripheral blood mononuclear cells (PBMC) and leukocyte functional properties. Single cells were identified by forward scatter (FSC)-height (H)/FSC-area (A) gating and the total population of leukocytes was visualized by side scatter (SSC)-A/FSC-A gating. Based on the distribution of cells according to their size and granularity, PBMCs and polymorphonuclear cells (PMN) were characterized **(A)**. Blood samples were stained using a combination of CD4/FITC and CD8/PE **(B)** or CD14/FITC and CD21/PE antibodies **(C)**. To determine the phagocytic activity of PMN and PBMC, samples were incubated with FITC-coupled *E. coli* and cellular intake of bacteria was measured by green fluorescence **(D)**. Formation of intracellular reactive oxygen species (ROS) was evaluated in samples stained with dihydrorhodamine (DHR) with or without stimulation with a phorbol ester. Representative image showed an overlay of ROS formation in unstimulated (light gray) and stimulated (dark gray) PMN **(E)**.

**Supplementary Table 1:** Effects of dietary L-carnitine supplementation from 1 day before until 14 days after intravenous LPS injection on red blood count and platelet-associated parameters of dairy cows. Measured with automated cell analyzer. Data are shown as least square means.

| **Days / Hours^1^** | **HCT^3^ [%]** | **MCV^4^ [fl]** | **MCHC^5^ [g/dl]** | **RDW^6^ [%CV]** | **PLT^7^ [10^3^/µl]** | **PCT^8^ [%]** | **MPV^9^ [fl]** | **PDW^10^ [%]** |
| --- | --- | --- | --- | --- | --- | --- | --- | --- |
| -1 | 24.5 | 47.9 | 33.6 | 16.29 | 470 | 0.144 | 3.16 | 19.1 |
| 0.5 | 26.2 | 48.2 | 33.3 | 16.31 | 441 | 0.122 | 2.95 | 19.6 |
| 1 | 25.1 | 48.3 | 33.2 | 16.25 | 407 | 0.119 | 3.05 | 18.8 |
| 2 | 23.8 | 48.3 | 33.2 | 16.29 | 376 | 0.117 | 3.17 | 18.0 |
| 3 | 24.0 | 48.3 | 33.6 | 16.33 | 373 | 0.113 | 3.10 | 18.8 |
| 4 | 24.5 | 48.1 | 33.5 | 16.36 | 377 | 0.119 | 3.23 | 19.0 |
| 6 | 25.0 | 48.0 | 33.5 | 16.28 | 362 | 0.111 | 3.09 | 18.8 |
| 9 | 25.4 | 48.3 | 33.4 | 16.25 | 423 | 0.115 | 2.89 | 19.6 |
| 12 | 24.9 | 48.0 | 33.5 | 16.29 | 394 | 0.109 | 2.92 | 19.3 |
| 24 | 25.1 | 48.0 | 33.4 | 16.30 | 408 | 0.120 | 3.08 | 18.7 |
| 48 | 24.5 | 48.0 | 33.5 | 16.42 | 436 | 0.124 | 2.95 | 19.3 |
| 72 | 24.1 | 48.0 | 33.5 | 16.40 | 443 | 0.131 | 3.04 | 18.7 |
| 7 | 24.0 | 47.8 | 33.6 | 16.34 | 599 | 0.173 | 2.93 | 19.2 |
| 14 | 23.8 | 48.1 | 33.8 | 16.51 | 534 | 0.147 | 2.84 | 19.8 |
| *p*-value |  |  |  |  |  |  |  |  |
| Group | 0.485 | 0.159 | 0.187 | 0.605 | 0.906 | 0.984 | 0.572 | 0.339 |
| Time | **<0.001** | **<0.001** | **0.001** | **<0.001** | **<0.001** | **<0.001** | **0.003** | **0.012** |
| Group x Time | 0.130 | 0.141 | 0.968 | 0.394 | 0.901 | 0.573 | 0.318 | 0.677 |
| PSE^2^ | 0.179 | 0.042 | 0.044 | 0.018 | 17.1 | 0.005 | 0.030 | 0.121 |

^1^ days / hours relative to LPS injection, ^2^ pooled standard error, ^3^ hematocrit, ^4^ mean corpuscular volume, ^5^ mean corpuscular hemoglobin concentration, ^6^ red cell distribution width, ^7^ platelets, ^8^ plateletcrit, ^9^ mean platelet volume, ^10^ platelet distribution width

**Supplementary** **Figure** **2:** Effects of dietary L-carnitine supplementation (control group = CON; carnitine group = CAR) from 1 day before until 14 days after intravenous LPS injection (red arrow) on derivates of hemoglobin of dairy cows. **(A)** percentage of carboxyhemoglobin (HGB), **(B)** percentages of deoxy-HGB, carboxy-HGB, met-HGB and oxy-HGB measured with automated blood gas analyzer. **(C)** Data statistics. Data are shown as least square means ± standard errors.


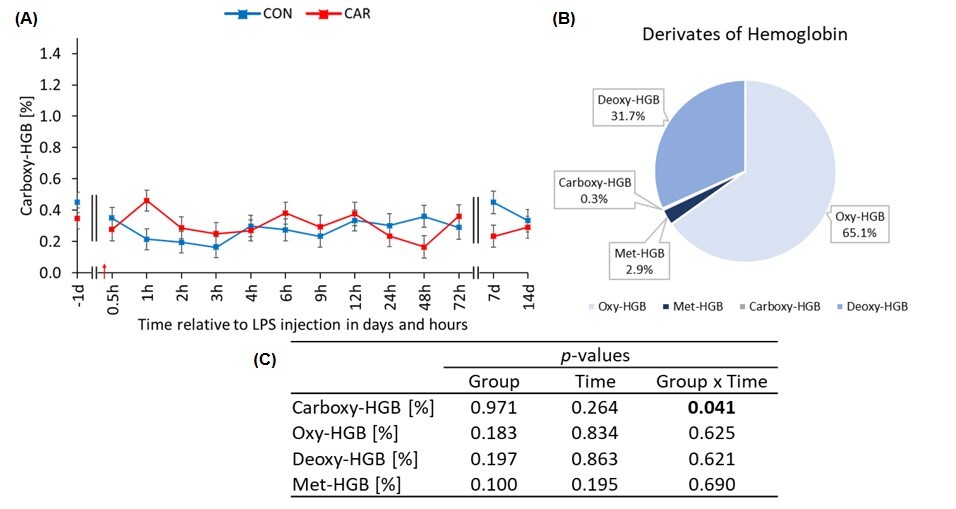


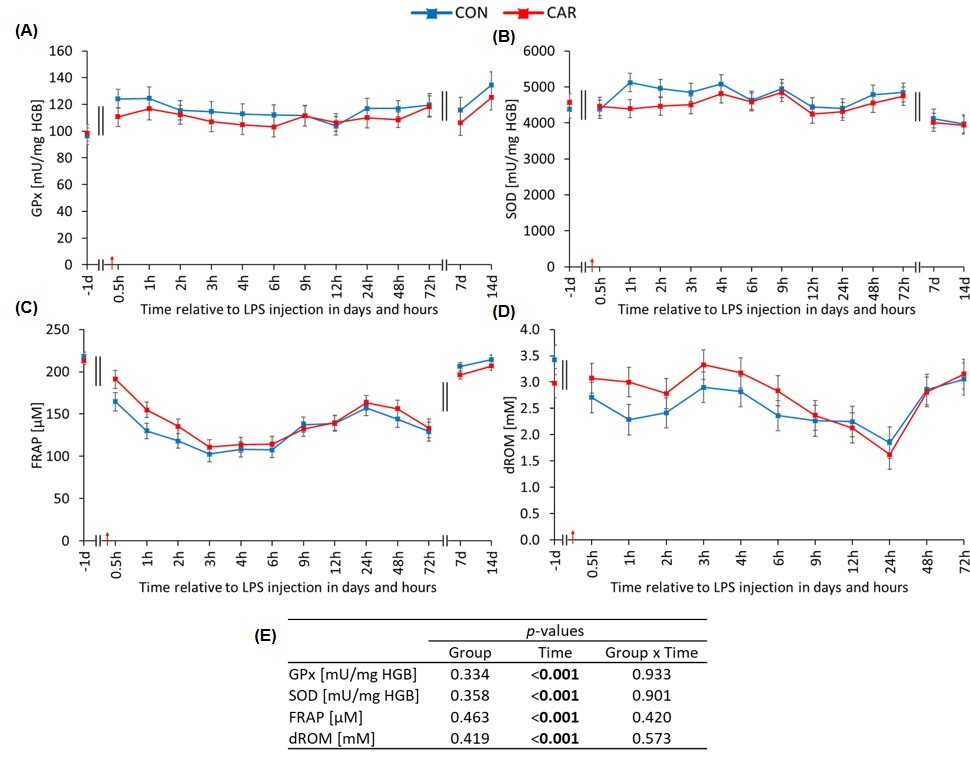
**Supplementary Figure 3:** Effects of dietary L-carnitine supplementation (control group = CON; carnitine group = CAR) from 1 day before until 14 days after intravenous LPS injection (red arrow) on indicators for the oxidative and antioxidative status of dairy cows. **(A)** glutathione peroxidase (GPx) activity measured in erythrocyte lysate, **(B)** superoxide dismutase (SOD) activity measured in erythrocyte lysate, **(C)** ferric reducing ability of plasma (FRAP), **(D)** derivates of reactive oxygen metabolites (dROM) measured in plasma. **(E)** Data statistics. Data are shown as least square means ± standard errors.

| **Days / Hours^1^** | **Eosinophils* [%]** | **Monocytes* [10^3^/µl]** | **Lymphocytes^#^ [%]** | **Monocytes^#^ [%]** |
| --- | --- | --- | --- | --- |
| -1 | 10.21 | 0.051 | 57.5 | 1.46 |
| 0.5 | 6.50 | 0.010 | 71.8 | 0.16 |
| 1 | 0.78 | 0.006 | 92.7 | 0.16 |
| 2 | 0.00 | 0.002 | 92.4 | 0.12 |
| 3 | 1.35 | 0.002 | 90.6 | 0.10 |
| 4 | 0.13 | 0.000 | 85.6 | 0.13 |
| 6 | 8.51 | 0.006 | 58.1 | 0.27 |
| 9 | 11.27 | 0.008 | 40.0 | 0.93 |
| 12 | 7.77 | 0.004 | 35.4 | 1.27 |
| 24 | 5.80 | 0.006 | 35.7 | 1.61 |
| 48 | 6.67 | 0.008 | 43.5 | 1.44 |
| 72 | 7.64 | 0.012 | 53.0 | 2.65 |
| 7 | 7.87 | 0.042 | 55.5 | 1.52 |
| 14 | 7.51 | 0.033 | 57.7 | 1.66 |
| *p*-value |  |  |  |  |
| Group | 0.143 | 0.760 | 0.710 | 0.088 |
| Time | **<0.001** | **<0.001** | **<0.001** | **<0.001** |
| Group x Time | 0.209 | 0.855 | 0.371 | 0.209 |
| PSE^2^ | 0.967 | 0.004 | 5.41 | 0.21 |

**Supplementary Table 2:** Effects of dietary L-carnitine supplementation from 1 day before until 14 days after intravenous LPS injection on white blood count of dairy cows. Data are shown as least square means.

^1^ days / hours relative to LPS injection, ^2^ pooled standard error, * measured with automated cell analyzer, ^#^ measured on blood smears

**Supplementary Table 3:** Effects of dietary L-carnitine supplementation from 1 day before until 14 days after intravenous LPS injection on functional properties of PMN^4^ of dairy cows. Measured by flow cytometry. Data are shown as least square means.

| **Days / Hours^1^** | **ROS^+3^ PMN^4^ unstimulated [10^3^/µl]** | **MFI^5^ of ROS^+3^ PMN^4^ unstimulated [x10^3^]** | **ROS^+3^ PMN^4^ stimulated [10^3^/µl]** | **MFI^5^ of ROS^+3^ PMN^4^ stimulated [x10^3^]** | **Stimulation Index ROS^+3^ PMN^4^** | **Stimulation Index MFI^5^ of ROS^+3^ PMN^4^** |
| --- | --- | --- | --- | --- | --- | --- |
| -1 | 0.312 | 10.7 | 3.93 | 91.6 | 18.6 | 9.3 |
| 0.5 | 0.172 | 9.6 | 1.24 | 91.5 | 8.7 | 10.2 |
| 1 | 0.149 | 9.6 | 0.32 | 79.0 | 2.2 | 8.7 |
| 2 | 0.090 | 12.3 | 0.24 | 89.9 | 2.5 | 7.7 |
| 3 | 0.082 | 13.1 | 0.30 | 94.2 | 3.0 | 7.9 |
| 4 | 0.079 | 10.0 | 0.40 | 93.8 | 4.2 | 10.2 |
| 6 | 0.132 | 8.5 | 1.51 | 105.2 | 13.7 | 14.0 |
| 9 | 0.099 | 7.6 | 3.24 | 109.8 | 53.0 | 16.5 |
| 12 | 0.078 | 8.3 | 4.28 | 93.9 | 105.2 | 13.8 |
| 24 | 0.117 | 12.2 | 7.52 | 111.6 | 91.6 | 10.8 |
| 48 | 0.230 | 10.7 | 6.29 | 100.9 | 46.2 | 10.5 |
| 72 | 0.263 | 10.0 | 4.77 | 105.3 | 24.3 | 11.4 |
| 7 | 0.195 | 10.6 | 4.25 | 78.2 | 31.7 | 8.0 |
| 14 | 0.291 | 12.1 | 3.76 | 84.1 | 19.7 | 7.4 |
| *p*-value |  |  |  |  |  |  |
| Group | 0.487 | 0.475 | 0.087 | 0.671 | 0.419 | 0.416 |
| Time | **<0.001** | **<0.001** | **<0.001** | **<0.001** | **<0.001** | **<0.001** |
| Group x Time | 0.109 | 0.639 | 0.455 | 0.547 | 0.642 | 0.548 |
| PSE^2^ | 0.021 | 0.42 | 0.61 | 0.018 | 8.5 | 0.7 |

^1^ days / hours relative to LPS-injection, ^2^ pooled standard error, **^3^** reactive oxygen species, ^4^ polymorphonuclear leukocytes, ^5^ mean fluorescence intensity

**Supplementary Figure 4:** Effects of dietary L-carnitine supplementation (control group = CON; carnitine group = CAR) from 1 day before until 14 days after intravenous LPS injection (red arrow) on functional properties of peripheral blood mononuclear cells (PBMC) of dairy cows. **(A)** percentage of unstimulated reactive oxygen species (ROS^+^) producing PBMC, **(B)** calculated absolute number of ROS^+^ PBMC, **(C)** percentage of 12-O-tetradecanoylphorbol-13-acetate (TPA)-stimulated ROS^+^ PBMC, **(D)** calculated absolute number of TPA-stimulated ROS^+^ PBMC, **(E)** mean fluorescence intensity (MFI) of unstimulated ROS^+^ PBMC, **(F)** MFI of TPA-stimulated ROS^+^ PBMC measured by flow cytometry. **(G)** calculated stimulation index (SI) of percentage of ROS^+^ PBMC, **(H)** SI of MFI of ROS^+^ PBMC. **(I)** Data statistics. Data are shown as least square means ± standard errors.


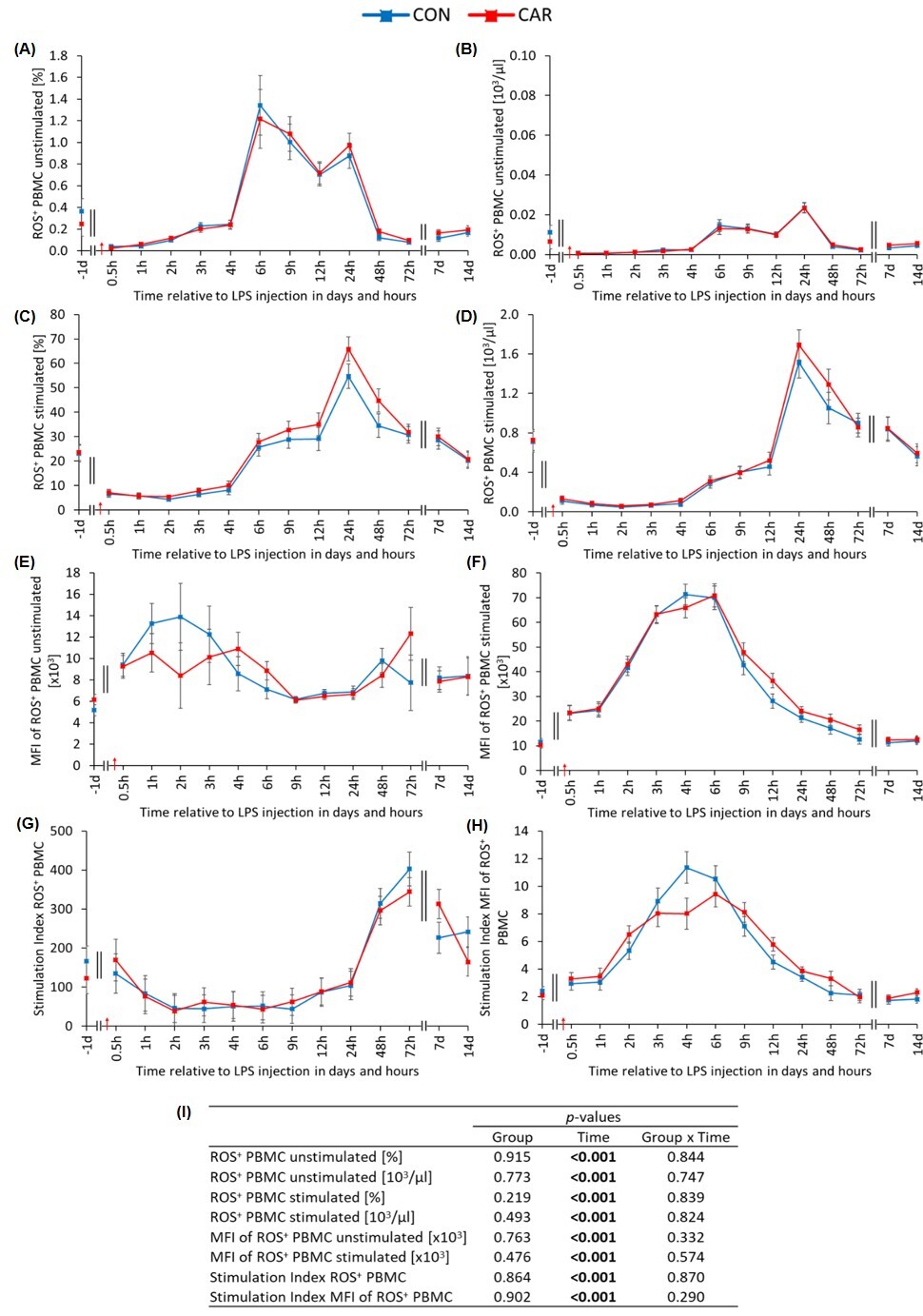


**Supplementary Table 4:** Effects of dietary L-carnitine supplementation from 1 day before until 14 days after intravenous LPS injection on phenotypes of leukocyte subsets of dairy cows. Measured by flow cytometry. Data are shown as least square means.

| **Days / Hours^1^** | **MFI^3^ of CD4^+^ [x10^3^]** | **MFI^3^ of CD8^+^ [x10^3^]** | **CD4/CD8 ratio** | **CD14^+^ [10^3^/µl]** | **CD21^+^ [%]** | **CD21^+^ [10^3^/µl]** | **MFI^3^ of CD21^+^ [x10^3^]** |
| --- | --- | --- | --- | --- | --- | --- | --- |
| -1 | 8.68 | 26.7 | 2.38 | 0.254 | 17.1 | 0.462 | 7.24 |
| 0.5 | 8.34 | 29.4 | 2.18 | 0.029 | 17.6 | 0.288 | 6.32 |
| 1 | 7.86 | 28.3 | 1.88 | 0.018 | 26.8 | 0.337 | 5.42 |
| 2 | 7.28 | 23.8 | 2.07 | 0.025 | 28.7 | 0.284 | 5.06 |
| 3 | 7.05 | 22.4 | 2.11 | 0.025 | 33.0 | 0.291 | 4.88 |
| 4 | 6.80 | 20.9 | 2.08 | 0.027 | 31.2 | 0.297 | 4.60 |
| 6 | 6.70 | 21.6 | 2.09 | 0.060 | 20.7 | 0.227 | 5.01 |
| 9 | 7.06 | 20.8 | 2.42 | 0.097 | 14.0 | 0.184 | 4.88 |
| 12 | 7.41 | 21.2 | 3.25 | 0.113 | 10.6 | 0.166 | 5.44 |
| 24 | 8.88 | 26.0 | 2.23 | 0.234 | 12.8 | 0.345 | 6.33 |
| 48 | 8.45 | 22.9 | 2.64 | 0.232 | 13.2 | 0.396 | 6.31 |
| 72 | 8.62 | 22.9 | 2.48 | 0.318 | 15.8 | 0.437 | 6.86 |
| 7 | 8.08 | 27.5 | 2.59 | 0.200 | 13.1 | 0.369 | 7.41 |
| 14 | 8.11 | 26.1 | 2.63 | 0.223 | 13.1 | 0.364 | 6.95 |
| *p*-value |  |  |  |  |  |  |  |
| Group | 0.122 | 0.995 | 0.350 | 0.921 | 0.170 | 0.412 | 0.268 |
| Time | **<0.001** | **<0.001** | **<0.001** | **0.001** | **<0.001** | **<0.001** | **<0.001** |
| Group x Time | 0.640 | 0.990 | 0.851 | 0.369 | 0.788 | 0.357 | 0.976 |
| PSE^2^ | 0.192 | 0.76 | 0.090 | 0.027 | 1.96 | 0.022 | 0.251 |

^1^ days / hours relative to LPS-injection, ^2^ pooled standard error, ^3^ mean fluorescence intensity, CD4^+^ = T-helper cells, CD8^+^ = cytotoxic T-cells, CD14^+^ = monocytes, CD21^+^ = B-cells

**Supplementary Table 5:** Effects of dietary L-carnitine supplementation from 1 day before until 14 days after intravenous LPS injection on clinical chemical parameters of dairy cows. Measured with an automated clinical chemistry analyzer. Data are shown as least square means.

| **Days / Hours^1^** | **Cholesterol [mmol/l]** | **γ-GT [U/l]** | **Bilirubin [mg/dl]** | **ALP [U/l]** | **ALT [U/l]** |
| --- | --- | --- | --- | --- | --- |
| -1 | 6.23 | 42.0 | 0.364 | 66.6 | 43.2 |
| 0.5 | 6.51 | 50.4 | 0.425 | 78.6 | 47.7 |
| 1 | 6.33 | 50.5 | 0.429 | 91.6 | 47.7 |
| 2 | 6.07 | 49.2 | 0.462 | 103.9 | 49.7 |
| 3 | 5.97 | 50.0 | 0.435 | 124.9 | 52.0 |
| 4 | 6.11 | 52.6 | 0.440 | 131.2 | 53.0 |
| 6 | 6.03 | 53.0 | 0.392 | 116.4 | 51.1 |
| 9 | 6.25 | 54.3 | 0.417 | 91.2 | 50.8 |
| 12 | 6.23 | 54.6 | 0.426 | 78.4 | 49.4 |
| 24 | 6.19 | 53.8 | 0.427 | 71.0 | 47.5 |
| 48 | 6.03 | 51.9 | 0.428 | 67.3 | 42.4 |
| 72 | 6.15 | 52.0 | 0.405 | 66.4 | 42.1 |
| 7 | 5.45 | 45.1 | 0.367 | 61.0 | 39.8 |
| 14 | 5.80 | 42.5 | 0.362 | 70.5 | 41.5 |
| *p*-value |  |  |  |  |  |
| Group | 0.062 | 0.947 | 0.196 | 0.614 | 0.893 |
| Time | **<0.001** | **<0.001** | **0.001** | **<0.001** | **<0.001** |
| Group x Time | 0.249 | 0.237 | 0.446 | 0.446 | 0.344 |
| PSE^2^ | 0.065 | 1.07 | 0.008 | 6.04 | 1.13 |

^1^ days / hours relative to LPS-injection, ^2^ pooled standard error, γ-GT = γ-glutamyltransferase, ALP = alkaline phosphatase, ALT = alanine aminotransferase
